# Endoscopic Activity Prediction in Ulcerative Colitis Using Hemogram-Derived Inflammatory Indices: Impact of Azathioprine Use on Diagnostic Performance

**DOI:** 10.3390/jcm15166464

**Published:** 2026-08-21

**Authors:** Muhammed Kaya, Gökhan Evren, İbrahim Durak, Tolga Düzenli, Hüseyin Köseoğlu, Mustafa Sadeçolak

**Affiliations:** 1 Department of Gastroenterology, Hitit University Corum Erol Olcok Training and Research Hospital, 19040 Corum, Türkiye; 2 Department of Nephrology, Faculty of Medicine, Uludağ University, 16240 Bursa, Türkiye; 3 Department of Gastroenterology, Faculty of Medicine, Hitit University, 19030 Corum, Türkiye; 4 Department of Gastroenterology, Faculty of Medicine, Samsun University, 55080 Samsun, Türkiye

**Keywords:** ulcerative colitis, endoscopic activity, Mayo score, Rachmilewitz EAI, inflammation, azathioprine

## Abstract

**Objective:** In ulcerative colitis (UC), endoscopic assessment remains central to disease monitoring, but repeated colonoscopy is invasive and resource-intensive. This study evaluated the association and discriminatory performance of the systemic immune–inflammation index (SII; platelet × neutrophil/lymphocyte) and the hemoglobin–albumin–lymphocyte–platelet (HALP; hemoglobin × albumin × lymphocyte/platelet) score for endoscopic activity in UC and explored whether azathioprine use modified these associations. **Methods:** Among 428 retrospectively reviewed patients with UC, 218 met the inclusion criteria. Mayo-defined activity (endoscopic score ≥ 2) and Rachmilewitz EAI-defined activity (score ≥ 4) were analyzed as separate outcomes. Laboratory parameters were compared between active disease and remission groups. Receiver operating characteristic (ROC) analyses, multivariable logistic regression, sensitivity analyses accounting for disease duration and treatment category, and azathioprine interaction analyses were performed. **Results:** According to the Mayo and Rachmilewitz EAI definitions, 80 (36.7%) and 150 (68.8%) patients, respectively, had active disease. For Mayo-defined activity, CRP showed the highest discrimination (AUC = 0.720), followed by SII (AUC = 0.692), whereas HALP showed limited discrimination (AUC = 0.598). In the primary multivariable Mayo model, CRP, SII, younger age, and male sex were independently associated with activity, whereas HALP and azathioprine use were not. Adding SII to CRP produced only a small numerical increase in AUC (0.720 to 0.752), and adding HALP produced essentially no further change. No significant interaction between azathioprine use and SII or HALP was identified. **Conclusion:** SII was associated with endoscopic activity but showed only modest discriminatory performance, while HALP had limited discriminatory and independent predictive value. CRP remained the strongest individual biomarker. SII and HALP should therefore be interpreted as adjunctive inflammatory indices rather than substitutes for established biomarkers or endoscopic assessment.

## 1. Introduction

Ulcerative colitis (UC) is known as a disease characterized by inflammation of the colonic mucosa, which can profoundly affect patients’ daily lives and progresses with periods of exacerbation and remission [1]. In diseases like UC that involve chronic inflammation in the body, the goal of treatment is not only to achieve clinical remission but also to achieve endoscopic and histological remission to reduce the ongoing inflammatory burden [2]. In recent years, approaches such as treat-to-target and The Selective Therapeutic Targets in Inflammatory Bowel Disease (STRIDE-II) have adopted longer-term treatment goals compared to traditional therapies in both Crohn’s disease and UC, emphasizing the need to achieve endoscopic remission of the disease [3,4,5]. This approach aims to protect patients from increased mortality, morbidity, and serious complications associated with inflammatory bowel diseases, such as colectomy.

Colonoscopy is a frequently used procedure considered the gold standard in the diagnosis of inflammatory bowel diseases, but due to its difficulty in application, the need for experience, its invasive nature, and its high cost, it is not always feasible to repeat it frequently [6]. Deciding on the need for treatment of subclinical mucosal inflammation, especially in patients with mild symptoms, is a challenging process for clinicians. Unfortunately, the role of non-specific inflammatory biomarkers such as C-reactive protein (CRP) and erythrocyte sedimentation rate (ESR) is insufficient, and access to more specific tests like fecal calprotectin is not available in every center [6,7,8]. Instead, the need for easily accessible but combined biomarkers has been frequently discussed in the literature in recent years for many diseases [9,10].

The systemic immune–inflammation index (SII), obtained by combining hemogram parameters, has been investigated across several inflammatory conditions as a marker reflecting the balance between circulating inflammatory and immune cell components [11]. In addition, it is argued that easily accessible and low-cost hematological parameters such as neutrophil-to-lymphocyte ratio (NLR) and platelet-to-lymphocyte ratio (PLR) scores may also reflect the acute attack phase of the disease in both inflammatory bowel diseases and some other internal diseases [12,13]. Apart from these, the HALP score, calculated using the formula hemoglobin × albumin × lymphocytes/platelets, and used particularly in cancer patients to demonstrate nutrition-related and some other immune parameters, is a potential biomarker that is increasingly being investigated [14,15].

In recent years, inexpensive and accessible inflammatory indices have gained increasing attention in the assessment of ulcerative colitis. Previous studies have reported associations between SII and UC activity, although the magnitude of discrimination varies across cohorts [16,17,18,19,20]. Xie et al. reported an AUC of 0.711 with an SII cut-off of 485.95 for active UC, while Lin et al. found AUCs of 0.647, 0.641, and 0.626 for SII, PLR, and NLR, respectively [18,21]. A recent meta-analysis including 1127 patients with UC reported pooled sensitivity of 0.622, specificity of 0.805, and a diagnostic odds ratio of 6.77 for SII, supporting an association with disease activity while also indicating imperfect diagnostic performance [22]. Evidence regarding HALP in inflammatory bowel disease remains considerably more limited [23]. Azathioprine may additionally affect hematologic components used to calculate these indices, making treatment-related confounding relevant when interpreting hemogram-derived markers.

In light of these considerations, this study aimed to evaluate the association and discriminatory performance of SII and HALP for endoscopic activity in UC, to compare their performance with conventional and simpler inflammatory markers, and to explore whether azathioprine use modified these associations.

## 2. Materials and Methods

### 2.1. Study Design and Participants

This retrospective study included adults (≥18 years) with UC followed at the Gastroenterology Clinic of a tertiary care center between 1 January 2019 and 1 December 2023. Patients were identified from the hospital information system and were eligible when an index colonoscopy performed at our institution had sufficient documentation to derive both endoscopic activity scores and concurrent laboratory data were available. Newly diagnosed patients and patients under long-term follow-up were not prospectively distinguished in the source records; therefore, this distinction could not be analyzed reliably.

Endoscopic activity was assessed using two established but structurally different scoring systems: the Mayo Endoscopic Score and the Rachmilewitz Endoscopic Activity Index (EAI) [24,25]. Given their different scoring structures, biomarker performance was analyzed separately using both the Mayo score and the more detailed Rachmilewitz EAI. A Mayo endoscopic score of 2–3 and a Rachmilewitz EAI score ≥ 4 were classified as active disease. Agreement between the dichotomized classifications was additionally evaluated. Endoscopic extent at the index colonoscopy was coded as 0 (no active endoscopic involvement), 1 (proctitis), 2 (left-sided colitis), or 3 (extensive colitis). Historical maximum Montreal extent at initial UC diagnosis was not consistently available; thus, category 0 is a study-specific code for absence of active involvement at the index examination and should not be interpreted as a formal Montreal E0 category [26].

Inclusion criteria were age ≥ 18 years, a diagnosis of ulcerative colitis, availability of colonoscopy reports allowing assessment of both the Mayo Endoscopic Score and the Rachmilewitz EAI, and availability of laboratory parameters obtained at the time of the index colonoscopy.

Exclusion criteria included missing or incomplete records; infectious, hematologic, oncologic, rheumatologic, or other inflammatory conditions that could affect inflammatory parameters; and use of systemic corticosteroids or biologic agents at the index assessment. Because of the retrospective study design, a standardized washout period for recently discontinued corticosteroids or biologic agents could not be confirmed.

Laboratory variables recorded at the index assessment included white blood cell count (WBC), hemoglobin (Hgb), platelet count (Plt), lymphocyte count (Lym), neutrophil count (Neu), mean corpuscular volume (MCV), mean platelet volume (MPV), plateletcrit (PCT), platelet distribution width (PDW), albumin, total protein, ESR, and CRP. HALP was calculated as (Hgb × albumin × lymphocyte)/platelet, SII as platelet × neutrophil/lymphocyte, NLR as neutrophil/lymphocyte, and PLR as platelet/lymphocyte. Laboratory measurements were matched to the index colonoscopic assessment, and treatment category was recorded for that assessment. The retrospective records did not permit reliable determination of whether sampling occurred before or after a recent treatment initiation or escalation, nor could a uniform washout interval after recently discontinued therapies be verified.

Patients receiving azathioprine, either as monotherapy or with mesalazine, were classified as AZA(+); all others were classified as AZA(−). For treatment-adjusted sensitivity analyses, treatment was grouped as untreated, mesalazine-based therapy without azathioprine, or azathioprine-based therapy. Patients receiving systemic corticosteroids or biologic agents at the index assessment were excluded.

The two primary outcome variables were Mayo-defined endoscopic activity (score ≥ 2) and Rachmilewitz EAI-defined endoscopic activity (score ≥ 4), analyzed separately. The main predictor variables were SII and HALP. CRP and other hematologic parameters were evaluated as comparator markers. Age, sex, and azathioprine use were included as covariates in the primary multivariable models. Additional sensitivity analyses incorporated disease duration and treatment category (untreated, mesalazine-based therapy, or azathioprine-based therapy) when disease-duration data were available.

### 2.2. Sample Size Calculation

Based on a similar study conducted by Pakoz ZB et al. [19] involving a total of 81 patients, the required sample size was calculated using SII values between remission and active disease groups, with a power of 0.90 and a significance level of 0.05. Accordingly, it was determined that a total of 40 patients—20 in each group—would be sufficient. Similarly, using the data from the study conducted by Lin H et al. [18] on 187 patients, the required sample size was calculated with a power of 0.90 and a significance level of 0.05, indicating that a total of 148 patients—74 in each group—would be needed.

### 2.3. Statistical Analysis

Statistical analyses were performed using IBM SPSS Statistics for Windows, Version 27.0 (IBM Corp., Armonk, NY, USA), with additional prespecified re-analyses performed for the revision. Continuous variables were summarized as mean ± standard deviation or median (Q1–Q3), as appropriate. Normality was assessed using Kolmogorov–Smirnov and Shapiro–Wilk tests, and between-group comparisons were performed using the Mann–Whitney U test. Differences across current endoscopic extent categories were explored using the Kruskal–Wallis test. Agreement between Mayo- and Rachmilewitz-defined activity was assessed using Cohen’s kappa. Multivariable logistic regression models used the enter method; the primary models included SII, HALP, CRP, age, sex, and azathioprine use. Multicollinearity was assessed before model inclusion, and model fit was summarized using Nagelkerke R^2^. Sensitivity models additionally adjusted for disease duration and treatment category in patients with available disease-duration data.

Diagnostic performance was evaluated using ROC analysis. AUCs, 95% confidence intervals, Youden-derived cut-offs, sensitivity, and specificity were calculated for CRP, SII, and HALP; NLR, PLR, and albumin were additionally examined as comparators. Because HALP and albumin decrease with greater inflammatory/nutritional burden, their direction was inverted for ROC analysis where appropriate. Predictive values for CRP, SII, and HALP were calculated at hypothetical pre-test probabilities of 10%, 25%, 50%, and 75% using the sensitivity and specificity observed at the Mayo-based Youden cut-offs. Exploratory logistic models compared discrimination for CRP alone, CRP + SII, and CRP + SII + HALP. The paired AUC difference was evaluated by bootstrap resampling. Potential effect modification by azathioprine was examined using interaction terms between azathioprine and each index. Statistical significance was set at *p* < 0.05.

## 3. Results

### 3.1. Patient Characteristics

**Figure 1:**
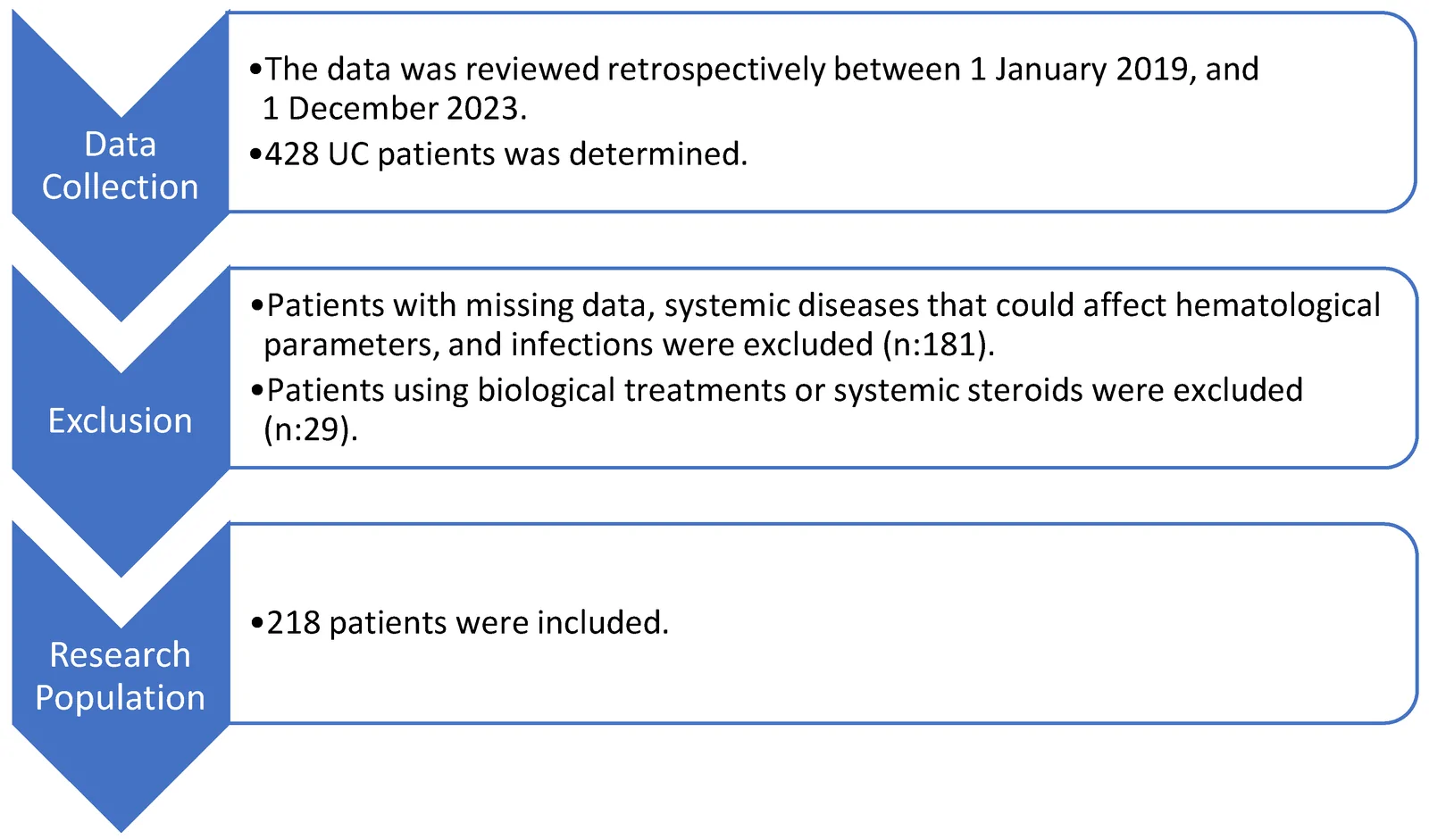
Study population flowchart.

Between the specified dates, 428 UC patients were identified, of whom 218 met the inclusion criteria and were included in the study. Demographic data and colonoscopic findings are presented in Table 1. The study flow is shown in Figure 1.

### 3.2. Laboratory Parameters According to Endoscopic Activity

In patients with active disease according to the Mayo score, CRP, WBC, neutrophil count, platelet count, PCT, and SII values were significantly higher compared with those in remission, while MCV and HALP scores were significantly lower (Table 2). Similarly, in patients classified as active according to the EAI, CRP, platelet count, WBC, SII, and PCT values were higher, whereas MCV and HALP scores were lower (Table 3).

### 3.3. Diagnostic Performance of Inflammatory Indices

**Figure 2:**
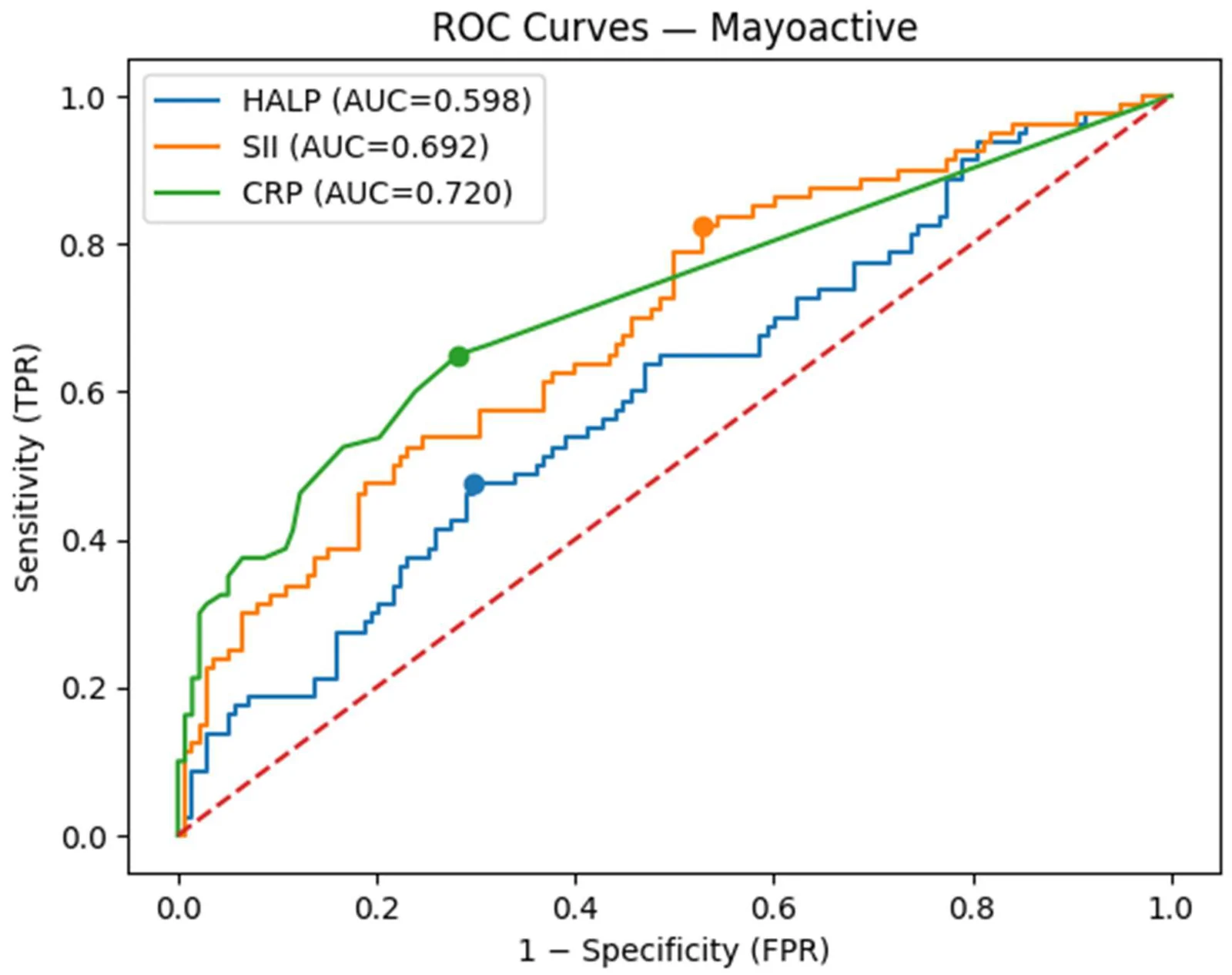
ROC curves for Mayo endoscopic activity (≥2): CRP, SII, and HALP.

In ROC analyses, the AUC value for predicting Mayo-defined endoscopic activity was found to be 0.720 (95% CI: 0.648–0.787) for CRP, 0.692 (95% CI: 0.617–0.762) for SII, and 0.598 (95% CI: 0.520–0.673) for HALP (Figure 2). The cut-off values determined according to the Youden index were 5.0 mg/L for CRP (sensitivity 65.0%, specificity 71.7%), 495.5 for SII (sensitivity 82.5%, specificity 47.1%), and 36.1 for HALP (sensitivity 47.5%, specificity 70.3%), respectively.

**Figure 3:**
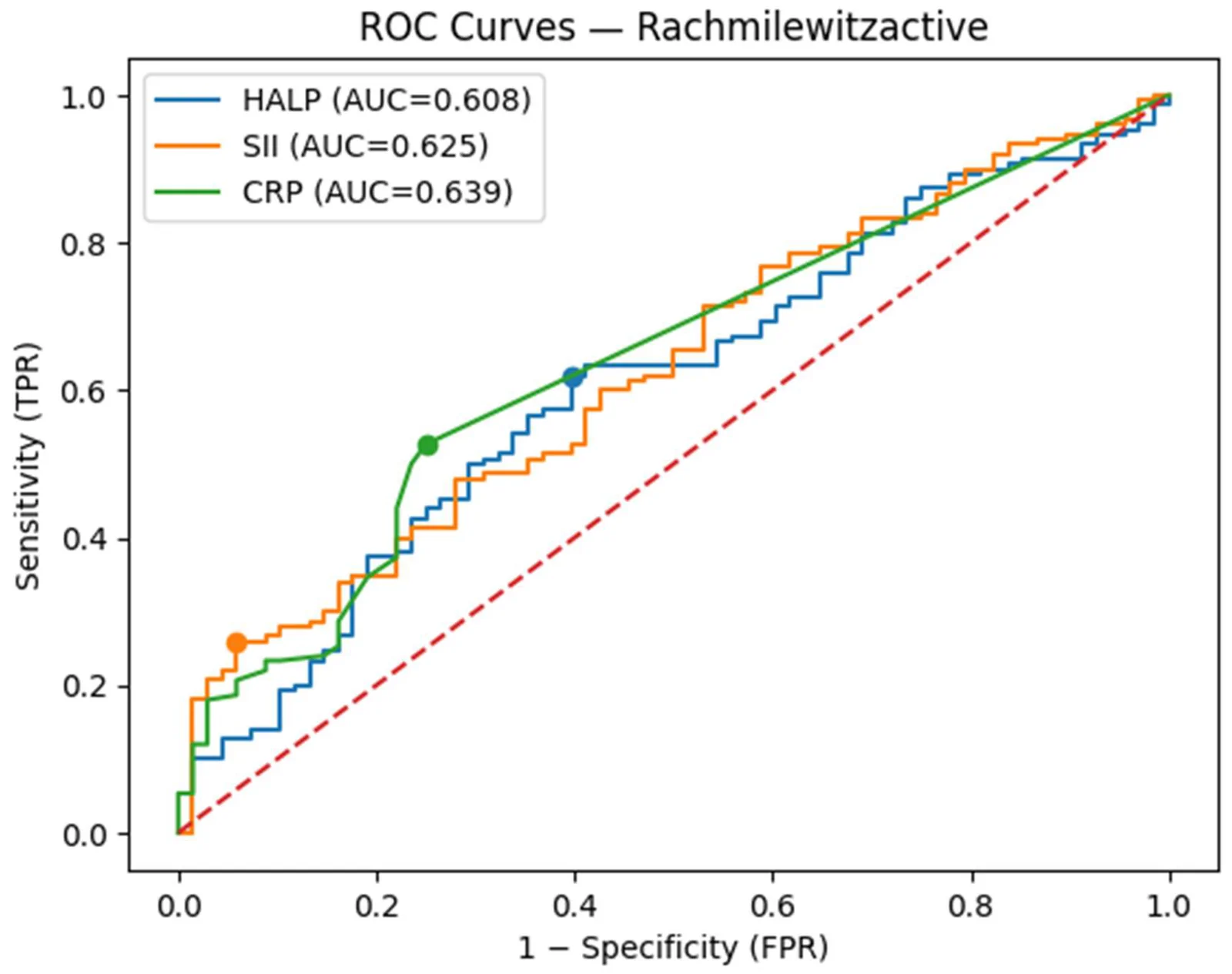
ROC curves for Rachmilewitz EAI (≥4): CRP, SII, and HALP.

AUC values for predicting Rachmilewitz EAI-defined endoscopic activity were found to be 0.639 (95% CI: 0.572–0.703) for CRP, 0.625 (95% CI: 0.550–0.699) for SII, and 0.608 (95% CI: 0.530–0.683) for HALP (Figure 3). The corresponding Youden cut-off values were 4.0 mg/L for CRP (sensitivity 52.7%, specificity 75.0%), 1049.5 for SII (sensitivity 26.0%, specificity 94.1%), and 47.2 for HALP (sensitivity 62.0%, specificity 60.3%).

### 3.4. Multivariable Analysis

In the primary multivariable model for Mayo-defined activity, higher CRP (OR = 1.072, 95% CI: 1.035–1.111; *p* < 0.001), higher SII (OR = 1.001, 95% CI: 1.000–1.001; *p* = 0.045), and male sex (OR = 2.434, 95% CI: 1.142–5.185; *p* = 0.021) were independently associated with active disease, whereas increasing age was inversely associated with activity (OR = 0.971, 95% CI: 0.951–0.991; *p* = 0.005). HALP and azathioprine use were not independently associated with Mayo-defined activity (Table 4). In the Rachmilewitz model, increasing age remained inversely associated with activity (OR = 0.981, 95% CI: 0.962–1.000; *p* = 0.047), while CRP showed a nonsignificant trend (*p* = 0.072); HALP, SII, sex, and azathioprine were not independently associated (Table 5). Nagelkerke R^2^ was 0.323 for the Mayo model and 0.128 for the Rachmilewitz model, indicating limited-to-moderate explanatory capacity, particularly for the latter outcome.

### 3.5. Additional and Sensitivity Analyses

**Figure 4:**
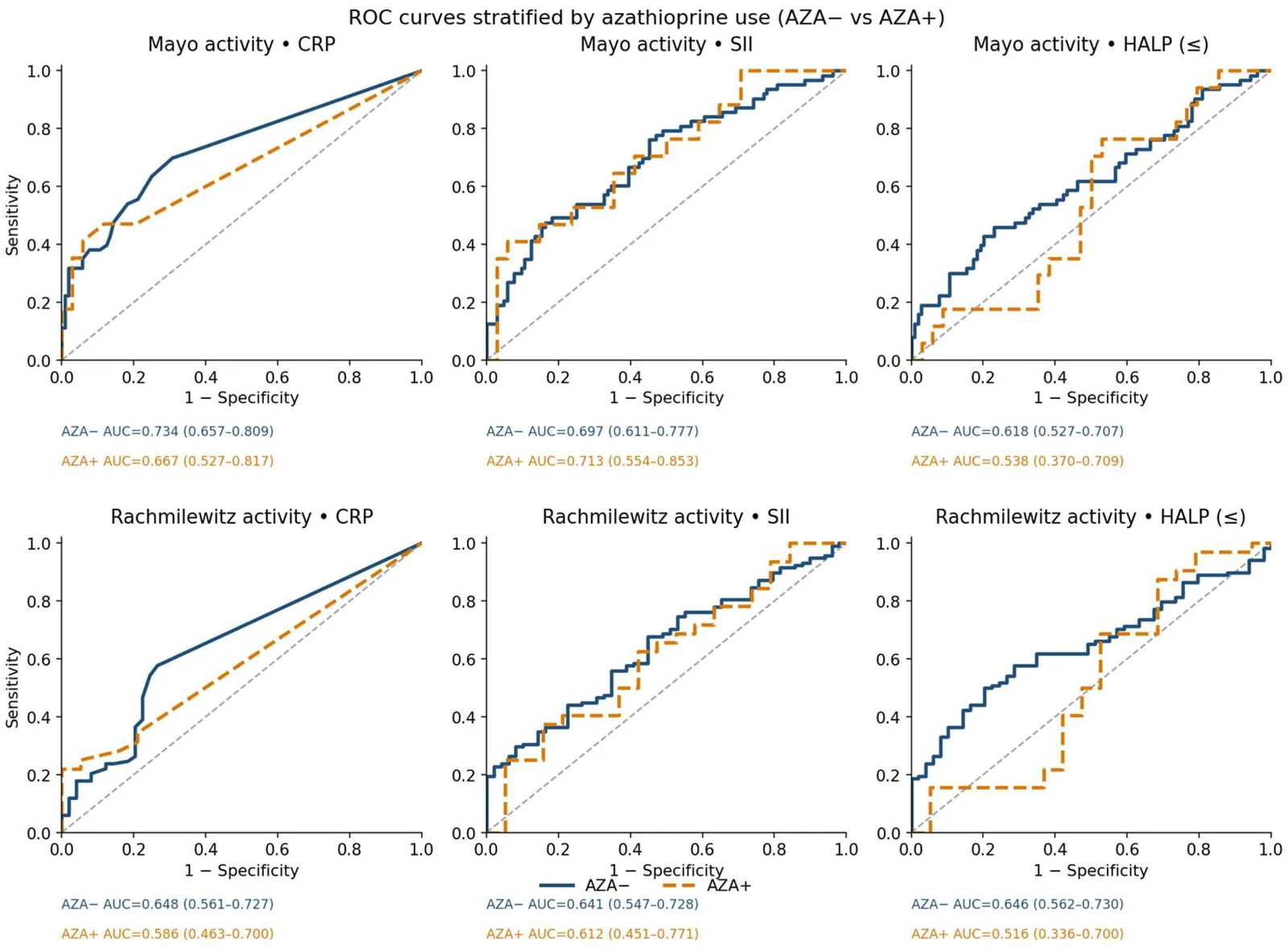
ROC curves for CRP, SII, and HALP stratified by azathioprine use (AZA− vs. AZA+) in Mayo- and Rachmilewitz-defined endoscopic activity. Stratified analyses descriptively examine biomarker discrimination according to azathioprine exposure; formal effect modification was assessed separately using interaction analyses.

For comparison with simpler hematologic ratios, the AUCs of NLR and PLR for Mayo-defined activity were 0.660 and 0.587, respectively, compared with 0.692 for SII and 0.598 for HALP; albumin alone showed limited discrimination (AUC = 0.546). For Rachmilewitz-defined activity, the corresponding AUCs were 0.570 for NLR, 0.602 for PLR, and 0.537 for albumin. In exploratory incremental analyses, adding SII to CRP produced a small numerical increase in AUC for Mayo-defined activity from 0.720 to 0.752; this difference was not statistically significant (bootstrap *p* = 0.180). Further addition of HALP produced essentially no change (AUC = 0.752). For Rachmilewitz-defined activity, the corresponding AUCs were 0.640, 0.652, and 0.651. Agreement between Mayo- and Rachmilewitz-defined activity was moderate (Cohen’s κ = 0.416): all 80 Mayo-active patients were also Rachmilewitz-active, whereas 70 Mayo-inactive patients were classified as active by the Rachmilewitz EAI. Among patients with active endoscopic involvement, CRP (*p* < 0.001), SII (*p* = 0.006), and NLR (*p* = 0.044) differed across extent categories, with the highest median CRP and SII values in extensive colitis; HALP, PLR, and albumin did not differ significantly. In a sensitivity analysis restricted to patients with available disease-duration data (n = 184), CRP (OR = 1.062, *p* = 0.002), SII (OR = 1.001, *p* = 0.014), and younger age (OR = 0.966, *p* = 0.004) remained associated with Mayo-defined activity after additional adjustment for disease duration and treatment category; disease duration and treatment category were not significant. For Rachmilewitz-defined activity, SII remained associated (OR = 1.001, *p* = 0.039), while the age association was attenuated (*p* = 0.064). Interaction analyses did not identify statistically significant modification of the SII- or HALP-activity associations by azathioprine use (all interaction *p* > 0.05). Predictive values at varying pre-test probabilities are presented in Table 6. The azathioprine-stratified ROC curves are shown in Figure 4.

## 4. Discussion

Our study evaluated whether routine and inexpensive blood-based inflammatory indices could provide useful information on endoscopic activity in patients with ulcerative colitis. Our findings suggest that hemogram-derived indices, particularly SII, show significant but modest associations with endoscopic activity in UC. SII was higher in patients with active disease, showed modest discriminatory performance for Mayo-defined activity (AUC = 0.692), and remained independently associated with activity in the primary and sensitivity analyses. HALP also differed between active and inactive disease in unadjusted analyses, although its discriminatory performance was lower and it did not remain independently associated after adjustment. Thus, these indices should not be viewed as substitutes for endoscopic assessment, but SII in particular may provide complementary information when interpreted together with established clinical and biochemical markers.

The STRIDE-II guideline, which provides comprehensive recommendations to clinicians regarding inflammatory bowel diseases, suggests a holistic assessment of biomarkers such as fecal calprotectin and CRP together with endoscopic activity findings to determine whether patients are achieving their expected treatment goals [3]. However, the infrequent repetition and invasive nature of endoscopic procedures highlight the need for additional non-invasive biomarkers in this field. Although various biomarkers have been proposed for the follow-up of UC, no single blood-based marker is sufficiently accurate to replace endoscopy. In this context, inexpensive indices derived from routine blood counts may be useful as supportive markers, particularly when their limitations are recognized.

SII, a biomarker calculated from hemogram parameters, has been increasingly used in the diagnosis and prognostic assessment of many diseases in recent years [19]. Its use has been supported by clinical studies in malignancies, coronary artery disease, appendicitis, vasculitis, pulmonary embolism, and cerebrovascular diseases [27,28]. In UC, previous studies have also reported an association between SII and disease activity. Xie et al. [21] reported an AUC of 0.711 with an SII cut-off of 485.95 for active UC, which is close to the AUC of 0.692 and cut-off of 495.5 observed for Mayo-defined activity in our cohort. Lin et al. [18] reported AUCs of 0.647 for SII, 0.641 for PLR, and 0.626 for NLR. In our study, the Mayo-based AUC of SII was numerically higher than those of NLR (0.660) and PLR (0.587). A recent meta-analysis including 1127 patients reported a pooled sensitivity of 0.622, specificity of 0.805, and diagnostic odds ratio of 6.77 for SII [22]. Taken together, these data support a reproducible association between SII and UC activity, while also showing that its standalone discriminatory ability is moderate rather than strong.

CRP showed the highest individual discriminatory performance in our cohort (AUC = 0.720). This finding is not unexpected, as CRP is an established inflammatory marker; however, CRP is also a nonspecific acute-phase reactant and may increase in many inflammatory and infectious conditions. We therefore explored whether SII might provide additional information when considered together with CRP. Adding SII to CRP resulted in a small numerical increase in the Mayo-based AUC from 0.720 to 0.752, although this difference was not statistically significant (*p* = 0.180). Further addition of HALP did not materially change the AUC. Accordingly, our results do not establish a significant incremental diagnostic benefit of SII beyond CRP, but the numerical change suggests that SII may reflect inflammatory information that is not completely identical to CRP. This possible complementary role should be evaluated in larger prospective cohorts rather than interpreted as evidence of superior diagnostic performance.

Another practical biomarker evaluated in our analyses is the HALP score, which, similar to SII, has been studied in the follow-up of several inflammatory diseases and malignancies [29]. Examples include malignancies of the urinary system, lungs, and gastrointestinal system [29]. In the current comparisons, the diagnostic performance of HALP (AUC = 0.598 for Mayo-defined activity) was lower than that of SII and CRP. Nevertheless, HALP differed significantly between active and inactive groups in the unadjusted Mayo analysis, suggesting that it may reflect changes accompanying inflammatory activity. Its association did not persist in the multivariable models, and albumin alone also showed limited discrimination (AUC = 0.546). Because HALP incorporates hemoglobin and albumin together with lymphocyte and platelet counts, it is influenced not only by inflammation but also by nutritional status, iron deficiency, protein loss, and other hematologic factors. These characteristics may explain why HALP was less specific for endoscopic inflammation in our cohort. The lower MCV observed in active disease may be compatible with iron-restricted erythropoiesis, although iron studies were not available and a specific mechanism cannot be established from our data. PCT and PDW were included as descriptive platelet indices; their findings were not interpreted as evidence of independent diagnostic utility.

Azathioprine is an immunomodulatory purine analog widely used in clinical practice to maintain remission in UC [30]. However, it can cause hematologic adverse effects such as thrombocytopenia, leukopenia, and pancytopenia through bone marrow suppression [31,32]. Therefore, its potential confounding effect is relevant in studies evaluating biomarkers derived from blood cell counts. In our study, azathioprine use was not independently associated with endoscopic activity, and no significant interaction was detected between AZA use and either SII or HALP. Sensitivity analyses that additionally accounted for treatment category did not materially change the main Mayo findings. These results suggest that the observed association between SII and endoscopic activity was not clearly modified by AZA exposure in this cohort. However, this finding should be interpreted cautiously because the AZA-treated subgroup was relatively small and information on dose, treatment duration, adherence, and recent treatment changes was not consistently available.

Another finding was that the biomarkers generally showed lower predictive performance for active disease defined using the Rachmilewitz Endoscopic Activity Index (EAI). The Mayo and Rachmilewitz systems are two established but structurally different approaches to endoscopic assessment: the Mayo score provides a relatively simple global grading, whereas the Rachmilewitz EAI incorporates a broader set of endoscopic features. Clinicians and studies may use either system, which was the reason for analyzing both definitions separately rather than combining them. As expected, the classifications were related, but agreement was only moderate (Cohen’s κ = 0.416). All patients classified as active by Mayo were also active according to Rachmilewitz, whereas 70 patients classified as inactive by Mayo met the Rachmilewitz activity threshold. The broader active group generated by the Rachmilewitz definition may partly explain the lower discriminatory performance observed for several biomarkers. These findings also support reporting the two analyses in parallel rather than treating the scoring systems as interchangeable.

Younger age was independently associated with Mayo-defined endoscopic activity and was also associated with Rachmilewitz-defined activity in the primary model. This finding is of interest, but it should be interpreted cautiously. Several studies have suggested that younger age in patients with ulcerative colitis is associated with greater disease activity and severity [33,34]. Younger patients may differ in disease phenotype, treatment exposure, duration of follow-up, or other unmeasured characteristics, and residual confounding cannot be excluded. In the sensitivity analysis including disease duration and treatment category, younger age remained associated with Mayo-defined activity, whereas the association was attenuated for Rachmilewitz-defined activity. Disease duration itself was not independently associated with activity in this analysis. Because the retrospective records did not allow reliable separation of newly diagnosed patients from those under long-term follow-up, the age finding should be regarded as hypothesis-generating rather than evidence of a direct biological effect.

The findings of this study should be interpreted in light of several methodological limitations. First, the retrospective and single-center design may increase the risk of selection bias and limit the generalizability of the findings. The study population included patients with different treatment exposures, while patients receiving systemic corticosteroids or biologic therapies at the index assessment were excluded. Moreover, the timing of recent treatment initiation, escalation, or discontinuation could not be reliably determined, and a standardized washout period for recently discontinued therapies could not be verified. Therefore, residual confounding related to treatment exposure cannot be completely excluded. In particular, the subgroup receiving azathioprine was relatively small (n = 51), and detailed information regarding drug dose, treatment duration, adherence, and recent treatment changes was not consistently available. Second, fecal calprotectin, an established non-invasive biomarker of intestinal inflammation in UC, was not consistently available and therefore could not be included for direct comparison. In addition, potentially relevant factors such as nutritional status and iron parameters were not comprehensively available. Finally, the analyses were based on laboratory measurements obtained at the index assessment rather than serial measurements; longitudinal changes in these indices may provide additional information regarding dynamic changes in endoscopic activity. These limitations should be considered when interpreting the diagnostic performance of the evaluated indices. Accordingly, our findings should be regarded as supportive and exploratory rather than as evidence for standalone clinical decision-making, and further validation in larger, prospective, multicenter cohorts incorporating established biomarkers such as fecal calprotectin is warranted.

The main strength of this study lies in its analysis of the relationship between routinely obtainable hemogram-derived inflammatory indices and endoscopic activity, an objective treatment target in UC. In addition to evaluating SII and HALP against two endoscopic scoring systems, the study compared these indices with CRP, NLR, PLR, and albumin; examined predictive values across different pre-test probabilities; and explored the potential influence of azathioprine and broader treatment category. SII showed a consistent association with Mayo-defined activity across the primary and sensitivity analyses and performed numerically better than the simpler NLR and PLRs, although its overall discrimination remained modest. These findings provide a clinically relevant but appropriately limited basis for further prospective evaluation of SII as an adjunctive marker.

## 5. Conclusions

Low-cost biomarkers derived from complete blood count and biochemical parameters may provide supportive information regarding endoscopic activity in UC. In our cohort, SII showed an association with disease activity, with modest discriminatory performance that was numerically higher than that of NLR and PLR, while HALP showed a weaker and less independent association. CRP remained the strongest individual marker among those evaluated. The small numerical increase in AUC when SII was added to CRP did not reach statistical significance and should be regarded as exploratory rather than confirmatory. These findings suggest that SII may hold potential as an adjunctive marker when interpreted alongside established clinical and biochemical findings. However, given their modest discriminatory performance, using SII or HALP for direct clinical decision-making at this stage may carry a risk of misclassifying disease activity, and their role should remain adjunctive rather than decisive. Neither marker should be used on its own or as a substitute for endoscopic assessment. Prospective studies including fecal calprotectin and broader treatment groups are needed to further clarify their potential clinical contribution.

## Figures and Tables

**Table 1:** Demographic and clinical characteristics of patients.

Variable	Total (n = 218)
Age (years), mean ± SD	47.2 ± 16.4
Female, n (%)	69 (31.7)
Male, n (%)	149 (68.3)
Disease duration (years), median (Q1–Q3)	3.0 (1.0–6.0)
Current endoscopic extent at index colonoscopy, n (%)	
No active endoscopic involvement	56 (25.7)
Proctitis	91 (41.7)
Left-sided colitis	52 (23.9)
Extensive colitis	19 (8.7)
Azathioprine use, n (%)	51 (23.4)
Mayo-active (≥2), n (%)	80 (36.7)
Rachmilewitz EAI—active (≥4), n (%)	150 (68.8)
Treatment category at index assessment, n (%)	
Untreated	44 (20.2)
Mesalazine-based therapy	123 (56.4)
Azathioprine-based therapy	51 (23.4)

**Table 2:** Laboratory parameters in active (≥2) and remission patients according to the Mayo score.

Parameter	Active (n = 80)	Remission (n = 138)	*p*
CRP (mg/L)	8.00 (3.00–24.25)	3.00 (3.00–5.00)	**<0.001**
SII	762.29 (540.59–1258.96)	542.48 (376.66–728.55)	**<0.001**
HALP	38.58 (27.72–55.83)	47.38 (34.57–59.30)	**0.016**
WBC (10^9^/L)	8.29 (6.77–10.77)	6.66 (5.62–8.29)	**<0.001**
Neutrophils (10^9^/L)	5.39 (3.91–7.46)	3.92 (3.16–5.56)	**<0.001**
Lymphocytes (10^9^/L)	1.97 (1.54–2.46)	1.89 (1.49–2.40)	0.553
Platelets (10^9^/L)	299.50 (244.50–346.00)	245.00 (209.50–301.25)	**<0.001**
Albumin (g/dL)	43.00 (40.00–45.00)	43.00 (41.00–46.00)	0.260
Hemoglobin (g/dL)	13.60 (12.30–15.03)	14.00 (12.60–15.10)	0.405
ESR (mm/h)	19.00 (8.00–39.00)	12.00 (7.00–27.00)	**0.024**
MCV (fL)	83.80 (79.53–86.80)	85.45 (82.90–88.85)	**0.003**
Plateletcrit (PCT)	0.30 (0.24–0.37)	0.25 (0.21–0.30)	**<0.001**
MPV (fL)	10.10 (9.47–10.62)	10.15 (9.60–10.78)	0.285
PDW	12.00 (10.67–13.12)	12.05 (10.93–14.88)	**0.039**
Total protein (g/dL)	73.00 (70.00–76.00)	73.50 (70.00–77.00)	0.395

Values are presented as median (Q1–Q3). Bold *p*-values indicate statistical significance (*p* < 0.05).

**Table 3:** Laboratory parameters in active (≥4) and remission patients according to the Rachmilewitz EAI score.

Parameter	Active (n = 150)	Remission (n = 68)	*p*
CRP (mg/L)	4.50 (3.00–9.75)	3.00 (3.00–3.25)	**<0.001**
SII	646.28 (444.10–1055.26)	542.48 (375.04–719.09)	**0.003**
HALP	39.92 (29.07–56.52)	48.84 (37.26–64.35)	**0.011**
WBC (10^9^/L)	7.61 (6.16–9.68)	6.57 (5.61–7.97)	**0.002**
Neutrophils (10^9^/L)	4.70 (3.52–6.40)	3.84 (3.25–5.65)	**0.007**
Lymphocytes (10^9^/L)	1.91 (1.50–2.44)	1.92 (1.53–2.35)	0.707
Platelets (10^9^/L)	283.00 (222.25–332.00)	243.50 (207.75–281.50)	**<0.001**
Albumin (g/dL)	43.00 (41.00–45.00)	43.50 (41.00–46.00)	0.381
Hemoglobin (g/dL)	13.85 (12.30–15.07)	13.75 (12.60–15.10)	0.472
ESR (mm/h)	16.00 (7.00–30.00)	12.00 (9.00–28.00)	0.659
MCV (fL)	84.30 (80.72–87.47)	86.85 (83.97–89.12)	**0.002**
Plateletcrit (PCT)	0.28 (0.23–0.34)	0.25 (0.21–0.29)	**0.003**
MPV (fL)	10.10 (9.50–10.70)	10.15 (9.60–10.80)	0.319
PDW	11.95 (10.72–13.97)	12.55 (10.95–14.53)	0.231
Total protein (g/dL)	73.00 (70.00–76.75)	74.00 (70.00–77.25)	0.421

Values are presented as median (Q1–Q3). Bold *p*-values indicate statistical significance (*p* < 0.05).

**Table 4:** Multivariable logistic regression—Mayo activity.

Variable	B	S.E.	Wald	*p*	Exp(B)	95% CI for Exp(B)
HALP	−0.004	0.009	0.21	0.649	0.996	0.979–1.013
SII	0.001	0.000	4.01	**0.045**	1.001	1.000–1.001
CRP	0.070	0.018	14.62	**<0.001**	1.072	1.035–1.111
Age	−0.030	0.011	7.97	**0.005**	0.971	0.951–0.991
Sex (male)	0.889	0.386	5.31	**0.021**	2.434	1.142–5.185
Azathioprine	−0.383	0.404	0.90	0.343	0.681	0.309–1.505
Constant	−0.711	0.723	0.97	0.326	0.491	0.119–2.028

Dependent variable: Mayoactive (0 = inactive, 1 = active), N = 218. Abbreviations: B, regression coefficient; S.E., standard error; Wald, Wald chi-square test; Exp(B), odds ratio; CI, confidence interval. All variables were entered simultaneously into the model (enter method). Bold *p*-values indicate statistical significance (*p* < 0.05).

**Table 5:** Multivariable logistic regression—Rachmilewitz EAI activity.

Variable	B	S.E.	Wald	*p*	Exp(B)	95% CI for Exp(B)
HALP	−0.005	0.008	0.36	0.549	0.995	0.980–1.011
SII	0.001	0.000	1.87	0.171	1.001	1.000–1.001
CRP	0.033	0.018	3.24	0.072	1.033	0.997–1.070
Age	−0.020	0.010	3.95	**0.047**	0.981	0.962–1.000
Sex (male)	0.493	0.348	2.00	0.157	1.637	0.827–3.242
Azathioprine	−0.516	0.363	2.02	0.155	0.597	0.293–1.217
Constant	1.082	0.721	2.26	0.133	2.951	0.719–12.115

Dependent variable: Rachmilewitzactive (0 = inactive, 1 = active), N = 218. Abbreviations: B, regression coefficient; S.E., standard error; Wald, Wald chi-square test; Exp(B), odds ratio; CI, confidence interval. All variables were entered simultaneously into the model (enter method). Bold *p*-values indicate statistical significance (*p* < 0.05).

**Table 6:** Predictive values of CRP, SII, and HALP for Mayo-defined endoscopic activity at different pre-test probabilities.

Pre-Test Probability	CRP PPV/NPV (%)	SII PPV/NPV (%)	HALP PPV/NPV (%)
10%	20.3/94.9	14.8/96.0	15.1/92.3
25%	43.4/86.0	34.2/89.0	34.8/80.1
50%	69.7/67.2	60.9/72.9	61.5/57.2
75%	87.3/40.6	82.4/47.3	82.8/30.9

PPV and NPV were calculated from the sensitivity and specificity observed at the Youden-derived Mayo cut-off for each biomarker. Abbreviations: PPV, positive predictive value; NPV, negative predictive value; CRP, C-reactive protein; SII, systemic immune–inflammation index; HALP, hemoglobin–albumin–lymphocyte–platelet score.

## Data Availability

The datasets generated and analyzed during the current study are available from the corresponding author on reasonable request.
